# Implications of Local Friendliness Violation for Quantum Causality

**DOI:** 10.3390/e23080925

**Published:** 2021-07-21

**Authors:** Eric G. Cavalcanti, Howard M. Wiseman

**Affiliations:** 1 Centre for Quantum Dynamics, Griffith University, Gold Coast, QLD 4222, Australia; 2 Centre for Quantum Dynamics, Griffith University, Brisbane, QLD 4111, Australia; h.wiseman@griffith.edu.au

**Keywords:** quantum foundations, quantum causality, causal models, quantum causal models, Wigner’s friend paradox, experimental metaphysics

## Abstract

We provide a new formulation of the Local Friendliness no-go theorem of Bong et al. [Nat. Phys. 16, 1199 (2020)] from fundamental causal principles, providing another perspective on how it puts strictly stronger bounds on quantum reality than Bell’s theorem. In particular, quantum causal models have been proposed as a way to maintain a peaceful coexistence between quantum mechanics and relativistic causality while respecting Leibniz’s methodological principle. This works for Bell’s theorem but does not work for the Local Friendliness no-go theorem, which considers an extended Wigner’s Friend scenario. More radical conceptual renewal is required; we suggest that cleaving to Leibniz’s principle requires extending relativity to events themselves.

## 1. Introduction

“For me … this is the real problem with quantum theory: the apparently essential conflict between any sharp formulation [of quantum theory] and fundamental relativity …. It may be that a real synthesis of quantum and relativity theories requires not just technical developments but radical conceptual renewal”.

(John S. Bell, 1984 [1]).

In a recent work [2], we (the present authors together with co-authors) proved what we called a “strong” no-go theorem on the Wigner’s friend paradox—the “Local Friendliness” (LF) no-go theorem. In this paper, we discuss further in what sense it is stronger than Bell’s theorem and give a broader picture of some of its implications. In particular, we discuss how it presents a challenge to a popular resolution of Bell’s theorem that goes back to Shimony [3].

Shimony proposed to separate Bell’s notion of Local Causality into two independent assumptions, which he called “Parameter Independence” (PI) and “Outcome Independence” (OI). His idea was that, while violation of PI was undoubtedly a case of action-at-a-distance—and was thus contrary to the letter of the theory of relativity—violation of OI was a far milder affliction—“passion at a distance” [3]—that was contrary only to the spirit of the theory of relativity. This allowed, the argument goes, for a “peaceful coexistence” [3,4,5] between quantum theory and relativity.

Shimony’s proposal has been criticized for missing the mark. For Bell, it left open the question of causal explanation of correlations [6]. The program of quantum causal models [7,8,9,10,11,12] has provided a candidate answer to this challenge by generalising the classical framework of causal models [13] to accommodate quantum correlations. This program shares important similarities with Shimony’s early proposal, and as we will show, is subject to the same challenge in light of the LF no-go theorem.

To better put these results and discussions in context, we present the Local Friendliness theorem within an updated version of the conceptual framework introduced by us in [14], where we reformulated the two theorems of John Bell (the 1964 [15] and 1976 [16] theorems – see Ref. [17] by one of us for a detailed discussion of the history and controversy surrounding the distinction.) in terms of fundamental metaphysical principles concerning events, space-time, and causality. This work was partly motivated by disagreements between two broad interpretational camps we referred to (following Reference [17]) as “realists” and “operationalists”. (As an aside: some whose positions are not in what we call the “realist” camp would nevertheless claim to be realists in some sense. We will keep the scare quotes when referring to these two camps here to avoid debates about the meaning of those terms.) Many of these disagreements arose from (often implicit) assumptions about the *meaning* of terms like “local”, and by using deeper principles, the foundations of these different perspectives could be made clearer. Moreover, we could present a “conciliatory” reformulation (Theorem 8 of [14]), in which the two camps could agree on the meaning of all the assumptions involved but only disagree about which assumptions were to be *rejected* in light of the theorem.

As we will discuss in detail in this paper, the new challenge alluded to in the opening paragraph here is a challenge for the operationalist camp. In our Bell-conciliation theorem [14], the “realist” camp would reject the principle of Relativistic Causality, while the “operationalist” camp would reject that of Decorrelating Explanation. This latter principle is the “quantitative” part of Reichenbach’s Principle of Common Cause (RPCC), which we split into two parts following the analysis proposed by EGC and Lal [8]. Rejection of this quantitative part of RPCC is also, implicitly or explicitly, the approach to resolve Bell’s theorem taken within the frameworks of quantum causal models [9,10,11,12]. However—and this is the new challenge—the assumption of Decorrelating Explanation is *not required* for the derivation of LF inequalities.

What are the implications of this? Firstly, there is of course the possibility that the LF inequalities derived in [2] fail to be violated with “genuine” observers (the “friends” of Wigner). We discuss this question at length in another paper [18]. For simplicity, here, we just acknowledge this as an open question and consider the implications of taking seriously the possibility that the LF violations demonstrated for very simple “observers” in recent experiments [2,19] can be maintained at the level of systems we may be strongly inclined to consider genuine observers—e.g., human-level artificial intelligences running in a very large quantum computer [18].

Assuming that nature violates Local Friendliness, then, this implies that a popular class of resolutions of Bell’s theorem—encompassing both “passion at a distance” and quantum causal models—is not available to resolve the LF theorem. Is a “peaceful coexistence” between quantum theory and relativity therefore impossible?

A “realist” would say yes. This camp would see the LF no-go theorem as a strengthening of the “nonlocal” position (rejecting Relativistic Causality), since it demonstrates more clearly than with Bell’s theorem the depth of the “essential conflict” between quantum theory and relativity, making it sharper than ever the need for “radical conceptual renewal”, in Bell’s words.

On the other hand, an “operationalist” may also find hope in a different sort of radical conceptual renewal. We will argue that Leibniz’s Principle of the Identity of Indiscernibles [20]—a methodological principle underlying Einstein’s principles of relativity and of equivalence, as well as the program of quantum causal models—can only be maintained by rejecting Absoluteness of Observed Events. This last is one of the assumptions of the LF no-go theorem, as well as a (typically implicit) assumption in Bell’s theorem. Maintaining Leibniz’s methodological principle, in other words, seems to require a kind of *strengthening* of relativity: that not only space-time but *events themselves* be described as relative.

This paper is organised as follows. In Section 2, we review Bell’s two theorems (from 1964 and 1976) and the implicit and explicit assumptions behind them. In Section 3, we break down Bell’s 1976 assumption of Local Causality into more primitive concepts to see how this can be given up while still holding to the existence of a Relativistic Causal Arrow, as Leibniz’s methodology requires. In Section 4, we show that this route, which is the one taken by quantum causal models, does not work when it comes to the LF theorem, because Local Causality is not part of that theorem. In Section 5, we break the assumptions down to the deepest principles, to display the options allowed by the theorems we consider. Critically, the LF theorem leaves fewer options, and we conclude in Section 6 by suggesting that cleaving to Leibniz’s principle requires rejecting Absoluteness of Observed Events.

## 2. Bell’s Theorem(s)

Bell’s theorem, and the LF theorem, are about quantum violations of different conjunctions of metaphysical assumptions through violations of constraints those assumptions imply for empirical correlations between events in certain experimental scenarios. Following [14], we start by stating two fundamental assumptions about events and space-time, typically left implicit in discussions of Bell’s theorem. In [14], we separated the various assumptions into “Axioms”, “Postulates” and “Principles”. The “Axioms” were so named because they were left as background assumptions in the statement of some or all of the theorems discussed, with their logical implications left implicit. This terminology is accurate for this section, but the LF theorem explicitly uses one of our Axioms in its formulation. Here and in subsequent definitions, whenever a word appears in small caps, it indicates a term whose meaning may be further specified or modified by other assumptions or principles.

**Axiom** **1** (Absoluteness of Observed Events)**.** *Every observed* event *is an absolute single* event*, not relative to anything or anyone.*

We called this Axiom “Macroreality” in [14], and it plays a crucial role in the LF theorem, as we will see.

**Axiom** **2** (Space-Time)**.** *Every* event *can be located in a background relativistic space-time, where concepts like past and future light-cone, space-like separation, etc., can be made experimentally well-defined.*

In [14], we called this assumption “Minkowski Space-Time”, but a flat space-time is not strictly required, only a time-orientable pseudo-Riemannian manifold.

In a Bell-type experiment, the observable events under consideration are choices of measurement settings (which we may label X,Y for the case of two distant parties), and their corresponding outcomes (which we may label A,B). We will use the same symbol (e.g., *A*) to refer interchangeably to a variable that ranges over possible values of this outcome and to the event corresponding to a particular outcome (say A=a) having been observed. We will thus talk about “correlations between events *A* and *B*” as a shorthand for “correlations between the variables associated with events *A* and *B*”.

The second of the two assumptions above, Space-Time, implies that those types of events can be located in space-time to sufficient precision in order to ascertain, e.g., that a pair of events (X,A) is contained in a space-like region separated from a region containing a pair of events (Y,B). The first assumption, Absoluteness of Observed Events, implies that these variables take well-defined values in every experimental run, and that it is therefore possible to define a conditional probability p(A,B|X,Y) for those events (which in quantum mechanics will be given by the Born rule).

Bell’s theorem demonstrates that certain phenomena predicted by quantum mechanics (i.e., the quantum predictions for certain sets of p(A,B|X,Y)) cannot be explained by models simultaneously satisfying certain sets of metaphysical assumptions. In our notation, Bell’s 1964 theorem can be expressed as:

**Theorem** **1** (Bell’s 1964 theorem)**.** *Quantum phenomena violate the conjunction of* No-Superdeterminism*,* Locality*, and* Predetermination *(together with AxiomS 1 and 2).*

These notions are precisely defined as follows.

**Principle** **1** (No-Superdeterminism)**.** *Any set of* events *on a space-like hypersurface (SLH) S can be taken to be uncorrelated with any set of interventions subsequent to S.*

This is a rigorous definition for the loose concept sometimes called “Freedom of Choice”, and what we call “interventions” here are usually called “free choices”. We prefer the term “intervention” here as it has a more precise meaning in the literature on causality [13,21]. That is, we emphasise that an intervention does not require the free will of a human agent, nor does it need to be itself entirely uncaused. The only important requirement is that an intervention can be chosen via external variables not a priori causally related with any of the other variables relevant to the experiment at hand.

**Principle** **2** (Locality)**.** *The probability of an observable* event *e is unchanged by conditioning on a space-like-separated intervention z, even if it is already conditioned on other* events *not in the future light-cone of z.*

This is a rigorous version of the concept called “Parameter Independence” by Shimony, also discussed (in the same year, and with much the same motivation) by Jarrett [22], who called it, as here, “Locality”. Bell’s use of this term in their 1964 theorem [15] also accords with this definition [17].

**Principle** **3** (Predetermination)**.** *Any observable* event *e is determined by a sufficient specification of* events *on any SLH S prior to e, possibly in conjunction with interventions subsequent to S.*

Here we again use the abstract noun employed by Bell in their 1964 paper [15], but note that “Determinism” is often also used for the same concept. It is also similar to the concept of “Outcome Determinism” used by Spekkens [23] in relation to contextuality, the main difference being that in discussions of contextuality relativistic space-time concepts typically do not play a role.

As an aside: here we are using definitions based on those in our earlier work [14], with changes in terminology or definitions noted and motivated when they arise, from Section 3 onwards. With regard the current section, we have realised that the definitions for No-Superdeterminism and Predetermination do not match well with natural language expectations when applied to models that require a preferred foliation, such as Bohm’s [24,25]. We will address that problem in a future publication. However, we note here that: (i) the theorems using these assumptions are still valid; (ii) Predetermination plays no part in the (more interesting) version of Bell’s theorem, from 1976, nor in the LF theorem; (iii) both No-Superdeterminism and Locality are replaceable by the single more natural assumption of Local Agency, as discussed in Section 5.

The celebrated Bell experiments of Aspect and co-workers [26,27] in the early 1980s led to almost universal acceptance of the veracity of the phenomena referred to in Bell’s theorem (Theorem 1). One of the initial responses [28] to this was to advocate giving up Predetermination (or “hidden variables” [28]), by pointing out that “standard quantum mechanics” was, after all, an indeterministic theory. Many still hold to this simple argument today. As far as Bell’s 1964 theorem alone is concerned, this could allow one to keep Locality, with the hope of thereby maintaining compatibility with relativity. This was, however, not satisfactory for Bell [6]:

“Do we then have to fall back on “no signalling faster than light" as the expression of the fundamental causal structure of contemporary theoretical physics? That is hard for me to accept. For one thing we have lost the idea that correlations can be explained, or at least this idea awaits reformulation”.

As Bell proved in 1976, the Bell inequalities can be derived from a principle specifying (what Bell took to be) a necessary condition for causal explanation in a relativistic space-time, the principle of Local Causality, which we reformulate as:

**Principle** **4** (Local Causality)**.** *If two space-like separated sets of* events **A** *and* **B** *are correlated, then there is a set of* events **C** *in the intersection of their past light cones such that conditioning on* **C** *eliminates the correlation.*

With this definition, Bell’s 1976 theorem can be formulated as follows.

**Theorem** **2** (Bell’s 1976 theorem)**.** *Quantum phenomena violate the conjunction of* No-Superdeterminism *and* Local Causality *(together with Axioms 1 and 2).*

**Figure 1:**
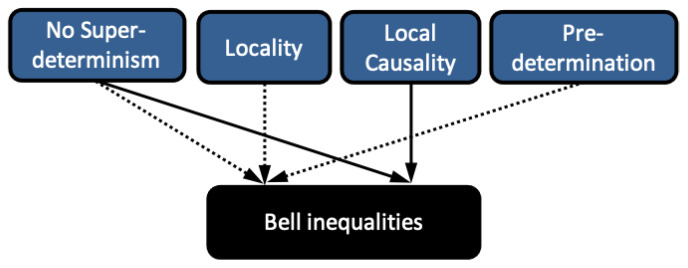
A graphical representation of Bell’s 1964 and 1976 theorems. Here and in subsequent figures, a concept is logically implied by the conjunction of its “parents” in the graph, i.e., all of those that have arrows pointing to it. A black box represents a concept that is known to be false (i.e., violated in nature). Bell’s 1964 theorem is indicated by the dotted arrows and Bell’s 1976 theorem by the full arrows.

The logical implications of Bell’s 1964 and 1976 theorems are illustrated in Figure 1. In light of Bell’s 1976 theorem, most physicists conclude (for different reasons, as we will discuss later) that it is Bell’s notion of Local Causality that needs to be rejected. That said, there are research programs pursuing theories violating No-Superdeterminism, and we note that the assumption of No-Superdeterminism given here can be violated by retrocausal [29,30]—as well as self-identifiedly superdeterministic [31,32]—approaches.

However, if Local Causality is rejected, how can we make sense of causal explanation of correlations in a relativistic space-time? We consider this question in the next section.

## 3. Classical and Quantum Causal Explanation

One of the basic principles of classical causal explanation was proposed by Hans Reichenbach in 1956. We reformulate it below, following [8,14].

**Principle** **5** (Reichenbach’s Principle of Common Cause (RPCC))**.** *If two sets of* events **A** *and* **B** *are correlated, and no* event *in either is a* cause *of any* event *in the other, then they have a set of common* causes **C***, such that conditioning on* **C** *eliminates the correlation.*

**Figure 2:**
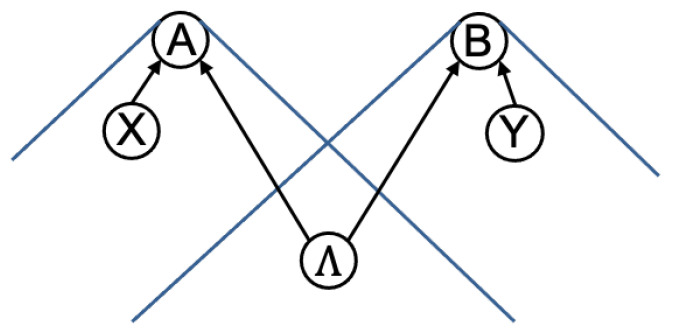
A directed acyclic graph (DAG) representing the causal structure of a Bell scenario involving two parties, Alice and Bob. *X* and *Y* represent Alice’s and Bob’s respective choices of setting, with *A* and *B* the corresponding outcomes. Λ represents a complete specification of the common causes between the two arms of the experiment. The DAG is motivated by the space-like separation between (X,A) and (Y,B), as indicated by the past light cones of *A* and *B*.

In more modern terms, Reichenbach’s Principle follows from a general framework of (classical) causal models [13] (An introduction to the classical causal model framework applied to quantum foundations can be found in [33].) In this framework, causal structure is represented by a directed acyclic graph (DAG), with random variables of interest associated with nodes, and arrows between nodes representing an asymmetric cause–effect relationship. The added requirement of acyclicity is intended to exclude causal loops. For example, the causal structure used in the derivation of a Bell inequality in a bipartite scenario has the form shown in Figure 2.

In a classical causal model, any probability distribution over the node variables that is compatible with a given graph must satisfy a constraint called the *Causal Markov Condition* (CMC). This can be expressed as the requirement that a variable *X* is independent of its non-effects Nd(X) (any nodes that are not among its “descendants” in the graph), conditional on its direct causes Pa(X) (its “parent” nodes). That is,

(1) p(X|Nd(X),Pa(X))=p(X|Pa(X)).

The Causal Markov Condition implies Reichenbach’s Principle of Common Cause as a special case [34]. The converse, however, does not hold. To see this, consider a causal graph with three nodes in a chain, X→Y→Z. The CMC implies that the middle node screens off the end nodes, i.e., p(Z|Y,X)=p(Z|Y). RPCC does not imply this condition.

The classical causal model framework (as the formulation of RPCC above) does not require any a priori assumptions about the causal structure in a Bell scenario. It allows, for example, that the causal structure may include a direct causal connection between a choice of setting (such as *X* in Figure 2) and a space-like separated outcome (such as *B*). To obtain Local Causality from this framework, it must be supplemented by some principle relating space-time and causal structure. It is easy to see that the following principle, taken in conjunction with RPCC, is sufficient to imply Local Causality:

**Principle** **6** (Relativistic Causal Arrow)**.** *Any* causes *of an* event *are in its past light-cone.*

We note that this concept was not explicitly defined in Reference [14] but, as will be discussed in Section 5, it is a simple consequence of deeper principles introduced there.

**Figure 3:**
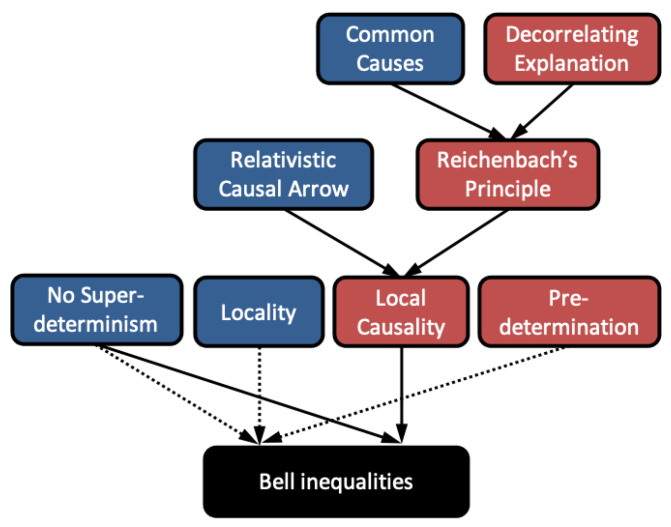
Local Causality is the conjunction of Relativistic Causal Arrow and Reichenbach’s principle. As discussed in the text, Leibniz’s Principle suggests the rejection Reichenbach’s principle, which is implied by the Principle of Common Cause and the Principle of Decorrelating Explanation. Quantum causal models resolve Bell’s theorem by rejecting all and only the concepts in red.

The relationships between the various concepts defined so far are represented in Figure 3. Thus, if in light of Bell’s 1976 theorem, one chooses to reject Local Causality, one is faced with a dilemma: reject Relativistic Causal Arrow or Reichenbach’s Principle of Common Cause.

### 3.1. Leibniz’s Principle and Causal Faithfulness

If one wishes to take the route of rejecting RPCC, the question becomes how to resolve the problem raised by Bell, that “we have lost the idea that correlations can be explained”. Why not give up Relativistic Causal Arrow instead, as is done, e.g., in Bohmian mechanics [24,25,35]? The reason why theories of this kind remain unattractive to a majority of physicists, we suggest, is that to maintain agreement with observations, they must necessarily violate, at a fundamentally hidden level, some of the operational symmetries we observe in the world. (This is not true if one considers a “non-equilibrium” version of Bohmian mechanics, which makes predictions different from those of operational quantum theory, as hypothesised by Valentini [36].)

More formally, we can say that theories rejecting Relativistic Causal Arrow fall foul of a principle that can be traced back to Leibniz—*Leibniz’s Principle of the Identity of Indiscernibles* [20]—according to which empirically indistinguishable scenarios should be represented by ontologically identical models. This is not to be thought of as a physical principle, but a methodological principle, like Occam’s razor, or a form of inference to the best explanation. In choosing between two empirically equivalent theories, we should prefer one that satisfies Leibniz’s Principle.

Spekkens recently argued [20] that Leibniz’s Principle can be identified as the guiding methodological principle underlying Einstein’s rationale for both the principle of relativity and the equivalence principle. In other words, to maintain the spirit of the theory of relativity, according to Spekkens, one should look for a theory that satisfies Leibniz’s Principle. However, how can it be done, if at all, in light of Bell’s theorem?

Firstly, let us consider: could there be a formulation of quantum theory that satisfies Leibniz’s Principle and Reichenbach’s Principle of Common Cause? An obvious candidate would be to reject No-Superdeterminism—but can a theory of this type maintain Leibniz’s principle?

If the purpose of rejecting No-Superdeterminism is to provide a locally *causal* explanation, then presumably one wants to maintain not only Reichenbach’s Principle of Common Cause, but the entire framework of classical causal models (otherwise the challenge becomes to replace that framework by some alternative).

Within that framework, one of us has argued [37] that Leibniz’s principle implies the principle of No Fine-Tuning, or Faithfulness. This principle requires that every conditional independence between variables (e.g., the no-signalling conditions) must arise as a consequence of the causal graph and not due to special choices of parameters in the model. In particular, therefore, if we cannot operationally signal faster than light, Leibniz’s principle suggests that we should prefer a theory that does not postulate faster-than-light-causation.

So could there be some faithful causal structure for quantum correlations, even if perhaps not the one implied by relativity? Unfortunately, as was shown in [33], no classical causal model can explain all instances of bipartite Bell inequality violations while satisfying Faithfulness. This includes even theories violating No-Superdeterminism, as long as they maintain the DAG structure of classical causal models and the Causal Markov Condition.

More recently, this result was generalised to arbitrary bipartite [37] and multipartite [38] Bell correlations, demonstrating that the relationship between fine-tuning and Bell nonlocality is generic and not an artefact of the simplest scenarios. (It was also shown, in Refs. [37,38], that no classical causal model can explain violations of Kochen-Specker noncontextuality [39] inequalities without fine-tuning. However, as discussed in [38], this requires a stronger notion of No Fine-Tuning, implicit in [37], but which is also motivated by Leibniz’s Principle.)

As argued in Refs. [37,38] (and similarly in Ref. [20]), these results suggest that to maintain Leibniz’s Principle, one must reject some of the basic assumptions of the framework of classical causal models, such as Reichenbach’s Principle of Common Cause.

### 3.2. “Peaceful Coexistence” through Quantum Causal Models?

As mentioned in the Introduction, Shimony’s proposal for “peaceful coexistence” did not satisfy Bell’s concerns about causal explanation. Drawing on the causal concepts discussed here so far, the difficulty is that, assuming the standard relativistic causal structure in Figure 2, both “Parameter Independence” and “Outcome Independence” follow from Reichenbach’s Principle of Common Cause.

In 2014, one of us and Lal [8] proposed to break Reichenbach’s Principle into two independent principles, which were formalised in Reference [14] as

**Principle** **7** (Principle of Common Cause)**.** *If two sets of* events *A and B are correlated, and no* event *in either is a* cause *of any* event *in the other, then they have a set of common* causes *C that* explains *the correlation.*

**Principle** **8** (Decorrelating Explanation)**.** *A set of* causes *C, common to two sets of events A and B,* explains *a correlation between them only if conditioning on C eliminates the correlation.*

Having made this division, we can ask: is it possible to maintain the Principle of Common Cause and replace Decorrelating Explanation (which was called “Factorization of Probabilities” in [8]) by some other principle of causal explanation in physics? In Reference [8], it was also suggested, following the framework of quantum conditional states of Leifer and Spekkens [7], that a candidate for such principle was the factorization of the Choi-Jamiolkowski operators corresponding to the quantum channels from the common cause *C* to Alice’s and Bob’s labs.

This suggestion was followed, in somewhat different ways, by various proposals for frameworks of quantum causal models [9,10,11,12], generalising the classical causal model formalism [13]. These proposals maintain the “qualitative” (in the terminology of [11]) part of Reichenbach’s Principle of Common Cause (the Principle of Common Cause above) while substituting the “quantitative” part (Decorrelating Explanation) by a suitable quantum generalisation thereof, to arrive at a quantum generalisation of Reichenbach’s Principle of Common Cause.

This approach to resolve the conflict between relativity and quantum theory is analogous to the route for “peaceful coexistence” proposed by Shimony [3], in that, when applied to a Bell scenario, quantum causal models satisfy Parameter Independence but not Outcome Independence. Nevertheless, unlike Shimony’s early proposal, the framework of quantum causal models meets Bell’s challenge for providing a (generalised notion of) causal explanation for quantum correlations. It also explains the usefulness of the classical framework, to which it reduces in the appropriate limits [12]. Furthermore, it can provide a faithful causal explanation of Bell correlations [10] and allows for causal discovery [10].

This is an interesting program, and it is making steps towards resolving what one of us called the “easy problem of Bell” [40], i.e., the problem of giving a causal explanation of Bell correlations. However, as previously argued by one of us in [40], quantum causal models (as currently formulated) cannot resolve the “hard problem of Bell”, namely the measurement problem. In the next Section 4, we provide a proof of this assertion, based on the Local Friendliness theorem [2].

## 4. The Local Friendliness Theorem

Wigner’s friend paradox is the quintessential intuition pump for the measurement problem. Some recent results have proposed no-go theorems based on extensions of the WFS including multiple observers and entanglement, such as the work by Frauchiger and Renner [41]. However, unlike Bell’s theorem, the Frauchiger-Renner theorem is not theory-independent—that is, it makes assumptions specifically related to quantum theory. (As an aside: more recent work generalises that theorem [42], highlighting the modal-logical nature of some of its assumptions. It could be perhaps said that the Frauchiger–Renner theorem is to the Kochen–Specker theorem [39] as the LF theorem is to Bell’s theorem, in the sense that the first two are expressed in terms of assumptions about (modal) logic, whereas the latter two are expressed in terms of metaphysical assumptions.) The Local Friendliness no-go theorem, by contrast, is theory-independent. It is based on the Extended Wigner’s Friend scenario introduced by Časlav Brukner [43,44].

**Figure 4:**
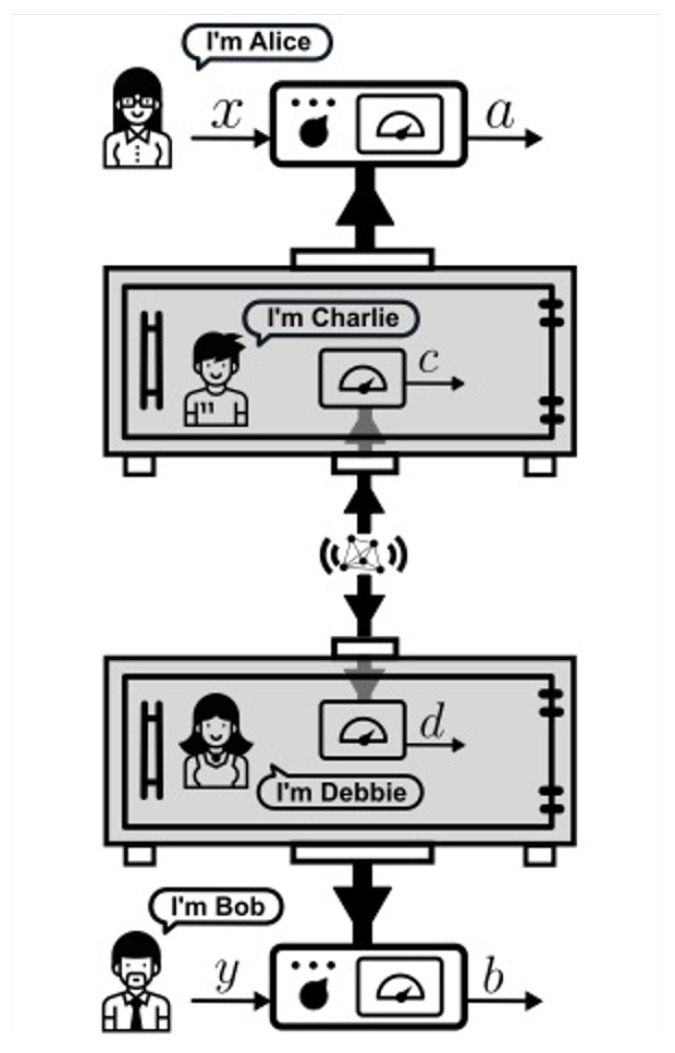
The Extended Wigner’s Friend Scenario, reproduced from Reference [2]. See text for details.

In this scenario, depicted in Figure 4, there are two labs, controlled by Alice and Bob, each containing a perfectly isolated vault, with a respective friend inside. The friends share a pair of particles prepared in an entangled state, on which they each perform a measurement on a fixed basis, obtaining outcomes *C* and *D*, respectively. Alice and Bob have choices of measurements labelled by *X* and *Y*, with respective outcomes *A* and *B*.

**Figure 5:**
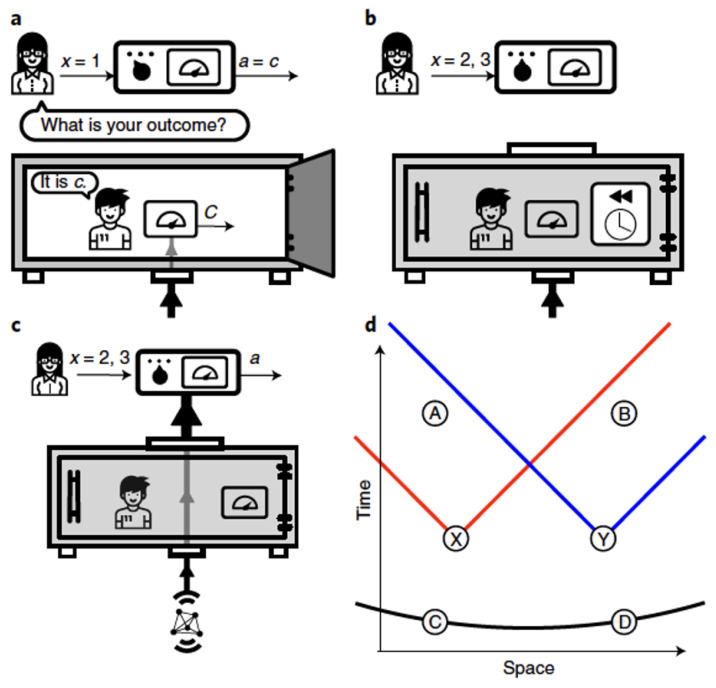
(**a**–**c**): The Local Friendliness protocol (see text for details). (**d**): A diagram of the space-time locations of the various events involved. (Reproduced from Ref. [2]).

In that protocol, as illustrated in Figure 5, if Alice chooses X=1 (Figure 5a), she opens the vault and asks Charlie what he observed and sets her own outcome to be equal to that of Charlie, i.e., A=C. If X≠1, she performs a measurement on the contents of the vault—including Charlie—in an incompatible basis. This can be done via reversing the unitary evolution that entangled Charlie (and his device, etc.) with his particle (Figure 5b) and proceeding to perform a measurement on the particle alone, corresponding to a different observable from that observed by Charlie (Figure 5c). A similar protocol is followed by Bob and Debbie.

For brevity, we will not review all of the details of the LF theorem here but refer the reader to Reference [2]. For the present purposes, the following summary is sufficient. We call Local Friendliness the conjunction of Locality, No-Superdeterminism and Absoluteness of Observed Events. We then show that in an Extended WFS, with a space-time arrangement of events as shown in Figure 5d, Local Friendliness implies constraints on the class of phenomena p(A,B|X,Y) that can be observed by Alice and Bob. These constraints can be put in the form of “LF inequalities”. We then show that LF inequalities can in principle be violated by quantum mechanics, if the requisite quantum operations can in principle be performed on observers. This leads to the following theorem:

**Theorem** **3** (Local Friendliness no-go theorem)**.** *Quantum phenomena violate the conjunction of* Absoluteness of Observed Events*,* Locality *and* No-Superdeterminism.

**Figure 6:**
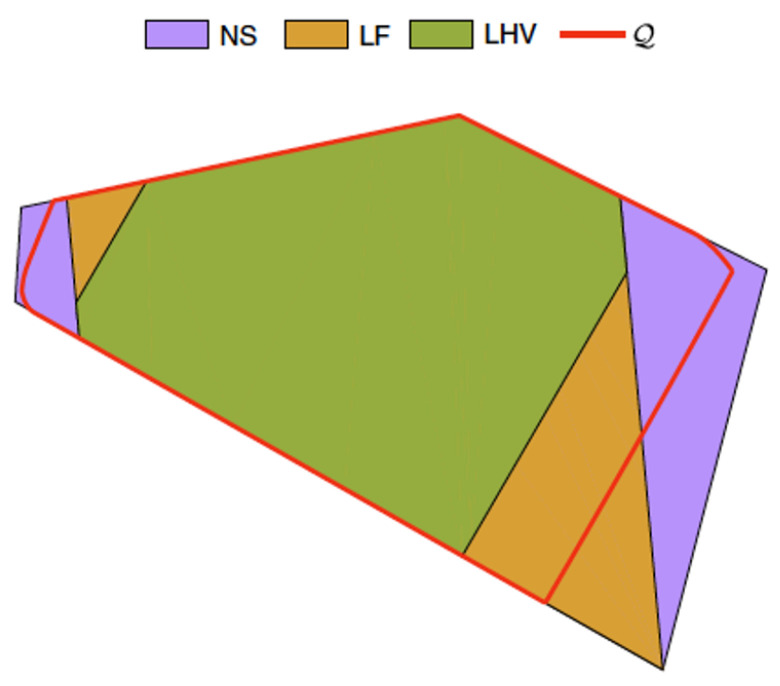
The Local Hidden Variable (LHV) polytope is the green area, the LF polytope is the orange area, and the grey polytope is the No-Signalling (NS) polytope. The red line is the boundary of quantum correlations. As the figure shows, the LF inequalities can be violated by quantum mechanics. See Reference [2] (from which the figure is reproduced) for further details.

As depicted in Figure 6, the set of LF correlations (which for a particular scenario has the form of a polytope—the “LF polytope”) strictly contains the Local Hidden Variable polytope (the set of correlations satisfying the Bell inequalities for a given scenario). In [2], we illustrated this hierarchy in a proof-of-principle experiment involving polarisation-entangled photons, with the path a photon takes playing the role of the “friend” and a polarising beam splitter the role of the observation. This hierarchy is a reflection of an important fact: the Local Friendliness assumptions are strictly weaker than the assumptions needed to derive a Bell inequality. This means that violation of LF inequalities has *strictly stronger implications* than violations of Bell inequalities.

A brief look at Figure 3 shows that it is *not* possible to resolve the LF theorem by dropping Decorrelating Explanation (and the other assumptions in red), as it was in the “peaceful coexistence” resolutions of Bell’s theorem—those assumptions are not used in the theorem. As prefigured, this undermines the narrative of the quantum causal models program, and related ideas (such as Shimony’s) that quantum mechanics and relativistic causality can be reconciled. To better understand what options for reconciliation remain open, we now turn to an analysis using deeper principles.

## 5. Bell and LF Theorems from More Fundamental Causal Principles

In this section, we clarify the implications of the LF theorem for quantum causality, and how it differs from Bell’s theorem, by refining many of the concepts introduced so far as consequences of more fundamental principles. Again, we largely follow [14], with some modifications noted along the way.

We first define the notion of causal past of an event as a set of events containing all of its causes.

**Definition** **1** (Causal Past)**.** *Any* cause *of an* event *is in its* causal past.

The next two principles impose spatio-temporal constraints on the causal past.

**Principle** **9** (Temporal Causal Arrow)**.** *For any* event *A, there is a space-like hypersurface S containing A that separates* events *in the* causal past *of A (on the same side of S as A’s past light-cone), from* events *that have A in their* causal past.

This principle is closely related to what we called Temporal Order in [14], except that here we use causal past instead of past, and further specify that it is in the same temporal direction as the past light-cone, to avoid a potential ambiguity. The following principle is a relaxation of the homonymous principle of Refence [14]:

**Principle** **10** (Relativistic Causality)**.** *The* causal past *of an* event *cannot be outside the light cones of that* event.

In [14], the principle of the same name specified that the past is the past light cone, but here the role of picking a direction of time for the causes is done by Temporal Causal Arrow instead. (The formulation we adopt here seems likely to be useful for disentangling retrocausal and superdeterministic approaches, which we will explore in future work.) In any case, the last two principles imply that the causal past is indeed in the past light cone. Thus, Temporal Causal Arrow and Relativistic Causality together imply Relativistic Causal Arrow.

Next we define the principle of Independent Interventions, a more cautious reformulation of “Free Choice” in Reference [14] (see [21] for a detailed discussion of the rationale for this criterion and the role it plays in a manipulability account of causation).

**Principle** **11** (Independent Interventions)**.** *An intervention has no relevant* causes *i.e., it can always be chosen via suitable variables that do not have* causes *among, nor share a common* cause *with, any of the other experimental variables.*

Similarly to how Relativistic Causal Arrow arises from the conjunction of two more fundamental principles, we also define a principle which arises from the conjunction of Independent Interventions and Principle of Common Cause. We call this Interventionist Causation (which in Reference [14] was called “Agent-Causation”):

**Principle** **12** (Interventionist Causation)**.** *If a set of relevant* events *A is correlated with an intervention, then that intervention is a* cause *of at least one* event *in A.*

**Figure 7:**
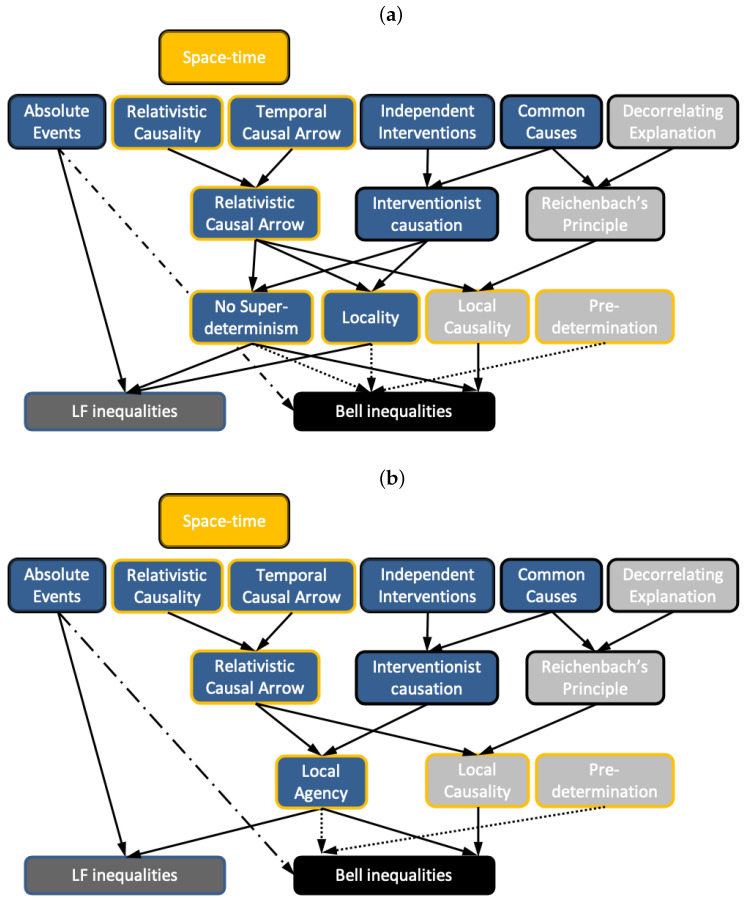
The full set of implications relevant for the Bell and LF theorems. The Bell inequalities can be derived from the conjunction of AOE (the dash-dotted line) together with either the conjunction of the dotted lines (Bell 1964) or the conjunction of the full black lines (Bell 1976). Principles that depend on spatio-temporal concepts assumed in the Space-Time Axiom are represented with a golden outline. In (**a**), we use the “traditional” shallow concepts of Locality and No-Superdeterminism, whereas in (**b**), we replace both of these by the deeper concept of Local Agency. Principles that are rejected within the proposals for “peaceful coexistence” between quantum and relativity theories outlined in Section 3.2 are represented by light grey boxes. Note that none of those are required to derive LF inequalities.

We now note that the conjunction of Relativistic Causal Arrow and Interventionist Causation imply both Locality and No-Superdeterminism, as depicted in Figure 7a. Indeed, that conjunction also implies the more natural concept of Local Agency [14].

**Principle** **13** (Local Agency)**.** *The only relevant* events *correlated with an intervention are in its future light cone.*

This can be used in place of Locality and No-Superdeterminism in both Bell’s 1964 theorem and the LF theorem, as discussed in References [2,14], respectively. In Bell’s 1976 theorem, Local Agency can be used to replace No-Superdeterminism. All of this is depicted in Figure 7b.

The violation of Bell inequalities thus requires the rejection of at least one of the deeper principles in the top row of Figure 7. In the case of the program for “peaceful coexistence” discussed in Section 3.2, resolution of Bell’s theorem is achieved by rejecting Decorrelating Explanation, and consequently all of the concepts shown in grey in Figure 7, while keeping all the remaining ones. However, the **main point of this paper** is that none of the concepts in grey in Figure 7 are required for deriving LF inequalities, and thus their rejection is not sufficient to resolve the LF no-go theorem. Only the blue assumptions go into the LF no-go theorem—in the simplest terms, only Absoluteness of Observed Events and Local Agency.

## 6. Discussion

Given the conclusion of the preceding section, what is the way forward for the program of quantum causal models? Firstly, it is important to point out that just as Bell’s theorem does not invalidate classical causal models as a useful tool in its regime of applicability, our results do not invalidate quantum causal models as a useful tool in *its* own regime of applicability—namely, in the description of the vast majority of quantum experiments where one can assume, for all practical purposes, a fixed Heisenberg cut—with “observers” on one side and “quantum systems” on the other.

One response could thus be to clearly state and accept the limited validity of each of these frameworks, rather than to attempt to resolve the conflict with the LF no-go theorem. This seems somewhat defeatist; a similar response in regards to classical causal models would have precluded the development of quantum causal models. A more interesting response could be to search for a further generalisation of quantum causal models to accommodate Wigner’s Friend scenarios.

### 6.1. Giving up Local Agency?

As we argued earlier in the paper, an underlying motivation for this program is to give a causal explanation for quantum correlations that satisfies Leibniz’s principle. Violation of Local Agency, on the other hand, seems to be in clear violation of this principle, insofar as we are not able to send signals outside of the future light cones of an intervention. However, let us consider whether this may be a too hasty conclusion.

Firstly, the program of quantum causal models has a clear aim of maintaining the Principle of Common Cause as a basic requirement for causal explanation. It also relies on an interventionist notion of causation for effective causal discovery and thus on the principle of Independent Interventions. It would also seem contrary to the aims of that program to reject Relativistic Causality providing a causal explanation compatible with relativity being one of its main aims.

Temporal Causal Arrow is required for obtaining a causal graph that has the form of a DAG, as is assumed in the initial formalisms for quantum causal models. On the other hand, the two most developed of these are based on the process matrix formalism, and as is well known, this formalism allows in general for causally non-separable processes, which are interpreted as representing situations with indefinite causal structure [45]. Indeed, recent work [46] considers an extension of the framework of quantum causal models of [11,12] to allow for cyclic causal graphs and show that this allows for a representation of certain classes of causally nonseparable processes, including some processes that violate causal inequalities as cyclic quantum causal models. However, there is no reason to think that postulating a causally nonseparable process would be sufficient to resolve the LF theorem, as we now explain.

The process matrix formalism [10,12,45] is limited to describing an experimental situation in terms of several labs receiving a quantum system as an input, upon which an instrument (represented as a set of CP maps that sum to a CPTP map) can be performed, the outcome of which (one of these CP maps) is denoted an “event”. After this CP map is applied to the system, it is sent through an output in the lab into the rest of the world, represented by the “process” to be potentially routed to the other labs, possibly in a causally nonseparable way. However, the agents, as well as the devices they use to perform their required instruments and obtain their corresponding outcomes, are left outside of the quantum description given by the process, just as in textbook operational quantum mechanics. In other words, the only thing that leaves the labs in that formalism is the quantum systems being measured by the various agents. In a Wigner’s Friend Scenario (WFS), however, *the entire contents of the friend’s lab* can be part of the input quantum system for a superobserver. This scenario simply cannot be described in current versions of the process matrix formalism.

Furthermore, one must recall that we require not only the violation of Temporal Causal Arrow, but that it is violated in such a way as to allow for the violation Local Agency, if this is to resolve the LF no-go theorem.

### 6.2. Giving up Absoluteness of Observed Events?

The remaining alternative is to give up Absoluteness of Observed Events. How does that fare as a path forward for the program of quantum causal models? We note from the discussion above that in quantum causal models, an “event” is the outcome of an instrument, associated with a CP map, and that the instrument is usually described as a classical variable, with a fixed “Heisenberg cut” applied in each lab. In a WFS, a fully quantum description of the “friends” and their labs is required instead. This suggests a direction to search for a generalisation of the process matrix formalism where the events observed in each lab can be described as *relative events*, where the outcomes of each instrument are encoded in relational variables associated with each lab, but which may not necessarily take well-defined values from a global perspective encompassing all of the labs. In such a formalism, there would not necessarily be a joint probability distribution over events observed in all labs, as usually assumed in the standard formalism.

In other words, if something like this suggestion is possible, the resolution of the conflict between quantum mechanics and relativity requires a *strengthening* of relativity: that not only space-time but *events themselves* be regarded as relative.

Absoluteness of Observed Events is rejected in some interpretations of quantum mechanics such as Everett [47], Relational QM [48] and QBism [49,50].

Everett [47] says (emphasis in the original), “One can arbitrarily choose a state for one subsystem, and be led to the relative state for the remainder. Thus, we are faced with a fundamental *relativity of states*, which is implied by the formalism of composite systems. It is meaningless to ask the absolute state of a subsystem—one can only ask the state relative to a given state of the remainder of the subsystem”, and later adds that “with each succeeding observation (or interaction), the observer state ‘branches’ into a number of different states. Each branch represents a different outcome of the measurement and the corresponding eigenstate for the object-system state.”

Relational QM is rooted in the work of Everett, but he does not subscribe to realism about the universal wave function. Instead, according to Laudisa and Rovelli [51]: “The world is therefore described by RQM as an evolving network of sparse relative *events*, described by punctual relative values of physical variables”.

QBism takes a more radical position, where a measurement outcome is a personal experience of an observer: In [49], Fuchs says: “What we learn from Wigner and their friend is that we all have truly private worlds in addition to our public worlds”. Furthermore, in [50], Mermin and Schack say: “What is real for an agent rests entirely on what that agent experiences, and different agents have different experiences”. A detailed discussion of the implications of the Local Friendliness no-go theorem for QBism is given in [52].

However, these accounts do not give a complete response to the challenge of providing a causal explanation that extends the classical framework. Whether this direction will work remains to be seen, but it certainly opens several questions. For example, if events are not absolute, then what is the meaning of the axiom of Space-Time? Would it need to also be generalised, perhaps by relaxing the assumption that there exists a single background space-time? Furthermore, if the motivation for rejecting Absoluteness of Observed Events is to keep Local Agency, what is the meaning of the notion of events required in that principle? We conjecture that Local Agency can be maintained at least in a suitably relaxed form, from the perspective of each agent. However, these are very challenging problems that are far beyond the scope of this paper. We also suggest that a fully satisfactory resolution of the measurement problem, underlying the LF no-go theorem, would require that these concepts either be ultimately explicable without direct reference to agents, or that they can be understood as describing an emergent level of description where these agent-centric concepts are applicable.

Whatever the solution, the implication is that something much more radical than we have been able to conceive so far is required for quantum causality to resolve the measurement problem in the form of the LF no-go theorem. We still need radical conceptual renewal.

## Data Availability

No new data were created or analyzed in this study. Data sharing is not applicable to this article.

## References

[1] Bell J.S. (1987). Speakable and Unspeakable in Quantum Mechanics. Introduction Remarks at Naples-Amalfi Meeting, 7 May 1984. Speakable and Unspeakable in Quantum Mechanics.

[2] Bong K.W., Utreras-Alarcón A., Ghafari F., Liang Y.C., Tischler N., Cavalcanti E.G., Pryde G.J., Wiseman H.M. (2020). A Strong No-Go Theorem on the Wigner’s Friend Paradox. Nat. Phys..

[3] Shimony A., Kamefuchi S. (1984). Controllable and Uncontrollable Non-Locality. Foundations of Quantum Mechanics in Light of New Technology.

[4] Myrvold W.C. (2002). On Peaceful Coexistence: Is the Collapse Postulate Incompatible with Relativity?. Stud. Hist. Philos. Sci. Part B Stud. Hist. Philos. Mod. Phys..

[5] Berkovitz J. (2007). Action at a Distance in Quantum Mechanics.

[6] Bell J.S. (1990). La Nouvelle Cuisine. Between Science and Technology.

[7] Leifer M.S., Spekkens R.W. (2013). Towards a Formulation of Quantum Theory as a Causally Neutral Theory of Bayesian Inference. Phys. Rev. A.

[8] Cavalcanti E.G., Lal R. (2014). On Modifications of Reichenbach’s Principle of Common Cause in Light of Bell’s Theorem. J. Phys. A Math. Theor..

[9] Pienaar J., Brukner C. (2015). A Graph-Separation Theorem for Quantum Causal Models. New J. Phys..

[10] Costa F., Shrapnel S. (2016). Quantum Causal Modelling. New J. Phys..

[11] Allen J.M.A., Barrett J., Horsman D.C., Lee C.M., Spekkens R.W. (2017). Quantum Common Causes and Quantum Causal Models. Phys. Rev. X.

[12] Barrett J., Lorenz R., Oreshkov O. (2019). Quantum Causal Models. arXiv.

[13] Pearl J. (2000). Causality: Models, Reasoning and Inference.

[14] Wiseman H.M., Cavalcanti E.G., Bertlmann R., Zeilinger A. (2017). Causarum Investigatio and the Two Bell’s Theorems of John Bell. Quantum [Un]Speakables II—Half a Century of Bell’s Theorem.

[15] Bell J.S. (1964). On the Einstein-Podolsky-Rosen Paradox. Physics.

[16] Bell J.S. (1976). The Theory of Local Beables. Epistemol. Lett..

[17] Wiseman H.M. (2014). The Two Bell’s Theorems of John Bell. J. Phys. A Math. Theor..

[18] Wiseman H.M., Cavalcanti E.G., Rieffel E.G. A thoughtful “Local Friendliness” no-go theorem.

[19] Proietti M., Pickston A., Graffitti F., Barrow P., Kundys D., Branciard C., Ringbauer M., Fedrizzi A. (2019). Experimental Test of Local Observer Independence. Sci. Adv..

[20] Spekkens R.W. (2019). The Ontological Identity of Empirical Indiscernibles: Leibniz’s Methodological Principle and Its Significance in the Work of Einstein. arXiv.

[21] Hausman D.M., Woodward J. (1999). Independence, Invariance and the Causal Markov Condition. Br. J. Philos Sci..

[22] Jarrett J.P. (1984). On the Physical Significance of the Locality Conditions in the Bell-Arguments. Nous.

[23] Spekkens R.W. (2005). Contextuality for Preparations, Transformations, and Unsharp Measurements. Phys. Rev. A.

[24] Bohm D. (1952). A Suggested Interpretation of the Quantum Theory in Terms of "Hidden" Variables. I. Phys. Rev..

[25] Bohm D. (1952). A Suggested Interpretation of the Quantum Theory in Terms of "Hidden" Variables. II. Phys. Rev..

[26] Aspect A., Grangier P., Roger G. (1981). Experimental Tests of Realistic Local Theories via Bell’s Theorem. Phys. Rev. Lett..

[27] Aspect A., Dalibard J., Roger G. (1982). Experimental Test of Bell’s Inequalities Using Time- Varying Analyzers. Phys. Rev. Lett..

[28] Rohrlich F. (1983). Facing Quantum Mechanical Reality. Science.

[29] Price H. (2008). Toy Models for Retrocausality. Stud. Hist. Philos. Sci. Part B Stud. Hist. Philos. Mod. Phys..

[30] Wharton K.B., Argaman N. (2020). Colloquium: Bell’s Theorem and Locally Mediated Reformulations of Quantum Mechanics. Rev. Mod. Phys..

[31] Hossenfelder S., Palmer T. (2020). Rethinking Superdeterminism. Front. Phys..

[32] Sen I., Valentini A. (2020). Superdeterministic Hidden-Variables Models I: Nonequilibrium and Signalling. Proc. Royal Soc. A.

[33] Wood C.J., Spekkens R.W. (2015). The Lesson of Causal Discovery Algorithms for Quantum Correlations: Causal Explanations of Bell-Inequality Violations Require Fine-Tuning. New J. Phys..

[34] Hitchcock C., Rédei M. (2020). Reichenbach’s Common Cause Principle.

[35] Maudlin T. (1994). Quantum Non-Locality and Relativity.

[36] Valentini A. (2010). Beyond the Quantum. Phys. World.

[37] Cavalcanti E.G. (2018). Classical Causal Models for Bell and Kochen-Specker Inequality Violations Require Fine-Tuning. Phys. Rev. X.

[38] Pearl J.C., Cavalcanti E.G. (2019). Classical Causal Models Cannot Faithfully Explain Bell Nonlocality or Kochen-Specker Contextuality in Arbitrary Scenarios. arXiv.

[39] Kochen S., Specker E.P. (1967). The Problem of Hidden Variables in Quantum Mechanics. J. Math. Mech..

[40] Cavalcanti E.G. (2016). Bell’s Theorem and the Measurement Problem: Reducing Two Mysteries to One?. J. Phys. Conf. Ser..

[41] Frauchiger D., Renner R. (2018). Quantum Theory Cannot Consistently Describe the Use of Itself. Nat. Commun..

[42] Nurgalieva N., del Rio L. (2019). Inadequacy of Modal Logic in Quantum Settings. Electron. Proc. Theor. Comput. Sci..

[43] Brukner C., Bertlmann R., Zeilinger A. (2017). On the Quantum Measurement Problem. Quantum [Un]Speakables II—Half a Century of Bell’s Theorem.

[44] Brukner C. (2018). A No-Go Theorem for Observer-Independent Facts. Entropy.

[45] Oreshkov O., Costa F., Brukner C. (2012). Quantum Correlations with No Causal Order. Nat. Commun..

[46] Barrett J., Lorenz R., Oreshkov O. (2021). Cyclic Quantum Causal Models. Nat. Commun..

[47] Everett H. (1957). “Relative state” formulation of quantum mechanics. Rev. Mod. Phys..

[48] Rovelli C. (1996). Relational quantum mechanics. Int. J. Theor. Phys..

[49] Fuchs C.A. Interview with a Quantum Bayesian 2012. https://arxiv.org/abs/1207.2141.

[50] Fuchs C.A., Mermin N.D., Schack R. (2014). An Introduction to QBism with an Application to the Locality of Quantum Mechanics. Am. J. Phys..

[51] Laudisa F., Rovelli C., Zalta E.N. (2021). Relational Quantum Mechanics, Spring 2021 edition.

[52] Cavalcanti E.G. (2021). The View from a Wigner Bubble. Found. Phys..

